# Monocular-Vision-Based Autonomous Hovering for a Miniature Flying Ball

**DOI:** 10.3390/s150613270

**Published:** 2015-06-05

**Authors:** Junqin Lin, Baoling Han, Qingsheng Luo

**Affiliations:** School of Mechanical Engineering, Beijing Institute of Technology, 5 Zhongguancun South Street, Haidian District, Beijing 100081, China; E-Mails linjunqin2010@163.com (J.L.); luoqsh@bit.edu.cn (Q.L.)

**Keywords:** monocular-vision sensor, vision measurement, flying height detecting, MAVs, hovering

## Abstract

This paper presents a method for detecting and controlling the autonomous hovering of a miniature flying ball (MFB) based on monocular vision. A camera is employed to estimate the three-dimensional position of the vehicle relative to the ground without auxiliary sensors, such as inertial measurement units (IMUs). An image of the ground captured by the camera mounted directly under the miniature flying ball is set as a reference. The position variations between the subsequent frames and the reference image are calculated by comparing their correspondence points. The Kalman filter is used to predict the position of the miniature flying ball to handle situations, such as a lost or wrong frame. Finally, a PID controller is designed, and the performance of the entire system is tested experimentally. The results show that the proposed method can keep the aircraft in a stable hover.

## 1. Introduction

The problem of hovering for a micro air vehicle (MAV) is considered here. It would sense the environment based on the information of the vision sensor, breaking the dependence on the Global Navigation Satellite Systems (GNSS), such as GPS and GLONASS, which will be selectively unavailable and will be disabled in cluttered or indoor environments. The commonly-used range measuring technologies to control the height in hovering, such as radar or laser range finder, are simply impractical for MAVs, because of the size and the weight barriers [1]. In addition to the small volume and light weight, the vision system also has advantages of passive illumination, low power consumption and low cost.

Many research works have been done on controlling the hovering of a vehicle based on vision. In the early years, helicopters were used in experiments because the MAV had not been widely studied. For example, a 67-kg Yamaha R-50 helicopter used a combination of stereo vision and feature tracking to control the hovering [2], and a fly-by-wire helicopter ATTHeS (Advanced Technology Testing Helicopter System) used the camera to track a two-dimensional template of *a priori* unknown features [3]. Both of them have the advantages of the sufficient space and carrying capacity of the helicopter. MAVs are characterized as small volume and limited payloads, so it is difficult to miniaturize the equipment for use on MAVs, such as the stereo vision needing a minimum baseline between two cameras.

Vision combined with structured environments has been applied successfully in hovering control [4,5,6]. For instance, an autonomous quadrotor aircraft can perform stable hovering above a pattern glyph with a data-fusion algorithm using both visual system measurements and inertial sensors [6]. However, the vision system relied on the pattern glyph, which is specially designed to align the aircraft with the glyph. Hence, these methods are still restrained by artificially structured environments in practical applications.

How to sense the height of MAVs by vision for hovering control using natural terrain is also a challenge. In Cherian’s work, height is measured by the machine learning to analyze the texture of the image captured by a downwards looking camera [7]. The reliance on the sufficient texture of images limits the application of this method. Optical flow can be used in several movement-control situations, such as terrain following [8], flight in the vicinity of obstacles or over flat terrain [9] and obstacle avoidance [10,11]. As for the control of hovering, optic flow is difficult with respect to measuring the height because it cannot produce a range unless there is a significant motion that goes against the objective of staying at a near perfect hover. Vision has achieved impressive results for the hovering of MAVs, which is fused with other sensors, especially the inertial measurement units (IMUs) [12,13,14]. These data fusion techniques are usually computationally complex, and the detecting reliability depends on several sensors.

While most of reviewed works have been done using vision assisted with other sensors, the vision-based hovering control proposed in this paper uses only one vision sensor. The MAV does not have any other sensors to measure its position or posture. Our method is inspired by honey bees, which utilize a stored snapshot as a visual marker and keep stable by the disparity between the current retinal image and the snapshot [15,16]. A camera is mounted directly under the MAV, and it takes an image of the ground underneath the MAV as a reference position. An error between the current positions and given reference position in the image plane is computed. When the height of the MFB changes, the distance of two points on the ground is invariable, but the distance of imaging points in the camera changes correspondingly. There is a negative correlation between these two distances. Therefore, we can achieve hovering control of the MFB by maintaining the distance variation near zero.

## 2. Vision-Based Position Estimation

For the purpose of achieving stable hovering, the positon of the MAV, which is relative to an external reference, such as the ground, should be determined at first. We estimate the position of the MAV based on visual information. In addition, a high-speed image processing algorithm also plays an important role to control the performance. Therefore, the image processing algorithm should be as fast as possible.

**Figure 1:**
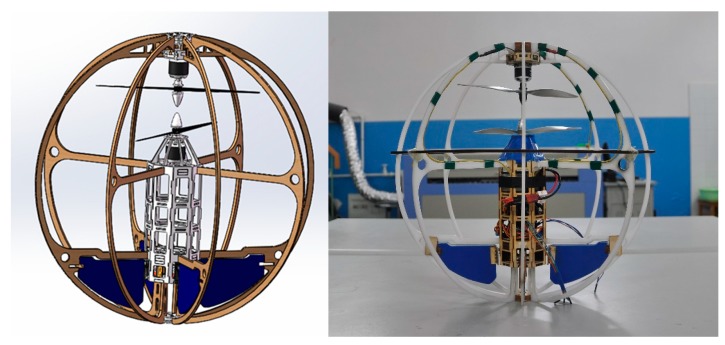
Miniature flying ball.

In this paper, we present an embedded MAV that uses information extracted from real-time images for hovering control. The ball-shaped outer shell of the MFB is lifted and propelled by the coaxial contra rotating twin rotors, which are mounted at the top of the ball (Figure 1). Four control surfaces at the button of the ball are used to control the motion direction of the ball freely. Therefore, the MFB almost has no restraints on its movement and can fly omnidirectionally. For the control of the hovering, the aircraft just needs the thrust of the coaxial contra rotating twin rotors, owing to its special power distribution, and it will always keep straight down to the ground.

### 2.1. Monocular Vision Analysis

**Figure 2:**
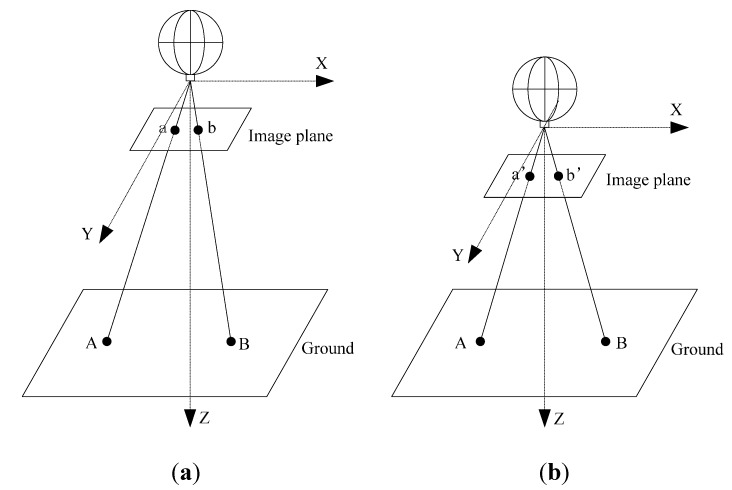
The monocular vision model and coordinate system used.

The camera is mounted directly under the MFB. In the camera coordinate system, the optical center of the camera is set as the origin, and the Z axis is parallel to the optical axis. The X and Y axes are parallel to the image plane, as shown in Figure 2a. A perspective projection model of a pinhole camera allows the position in a 2D image plane to be inferred from the 3D position [17]. The model projects an arbitrary point $A={\left(x_{w},y_{w},z_{w}\right)}^{T}$ on the ground to point $a={\left(u,v\right)}^{T}$ on the image plane (the camera image) expressed by Equation (1):

(1) $$z_{c}•\left[\begin{array}{c} u\\ v\\ 1 \end{array}\right]=\left[\begin{array}{cccc} \frac{f}{d_{x}} & -\frac{f}{d_{x}}\cot\theta & u_{0} & 0\\ 0 & \frac{f}{d_{y}\sin\theta } & v_{0} & 0\\ 0 & 0 & 1 & 0 \end{array}\right]\left[\begin{array}{cc} R & t\\ 0^{T} & 1 \end{array}\right]\left[\begin{array}{c} x_{w}\\ y_{w}\\ z_{w}\\ 1 \end{array}\right]$$

where $Z_{c}$ is an arbitrary positive scalar. The six intrinsic parameters ($f,d_{x},d_{y},\theta ,u_{0},v_{0}$) can be derived from the calibration of the camera. The extrinsic parameters, the R and T metrics, denote the rotation and translation of the camera. For the control of hovering, the aircraft just needs the thrust upward. It will always keep nearly straight down to the ground, and the image plane of the camera will keep approximately parallel to the ground. Therefore, the extrinsic parameters can be simplified as:

(2) $$R≐\left[\begin{array}{ccc} 1 & 0 & 0\\ 0 & 1 & 0\\ 0 & 0 & 1 \end{array}\right],T≐\left[\begin{array}{c} 0\\ 0\\ h \end{array}\right]$$

where h denotes the height of the camera. Then, we can get Equation (3):

(3) $$u-u_{0}=\frac{fx_{w}}{h},v-v_{0}=\frac{fy_{w}}{h}$$

Consider the situation of a line AB on the ground and the corresponding line ab on the image plane. When the height of the MFB changes into $h^{\prime }$, as shown in Figure 2b, ab on the image plane will change into $a^{\prime }b^{\prime }$:

(4) $$h=\frac{f•l_{AB}}{l_{ab}},h^{\prime }=\frac{f•l_{AB}}{l_{a^{\prime }b^{\prime }}}$$

From Equation (4), the measurement formula is obtained:

(5) $$\frac{h}{h^{\prime }}=\frac{l_{a^{\prime }b^{\prime }}}{l_{ab}}$$

The essential problem of the system is to achieve stable hovering of the MFB by maintaining $h^{\prime }=h$. According to Equation (5), this condition can be transferred into keeping $l_{a^{\prime }b^{\prime }}$ nearly equal to $l_{ab}$. Therefore, the problem of controlling the height of the MFB is turned into finding the corresponding points ab and $a^{\prime }b^{\prime }$.

### 2.2. Measurement from Images

**Figure 3:**
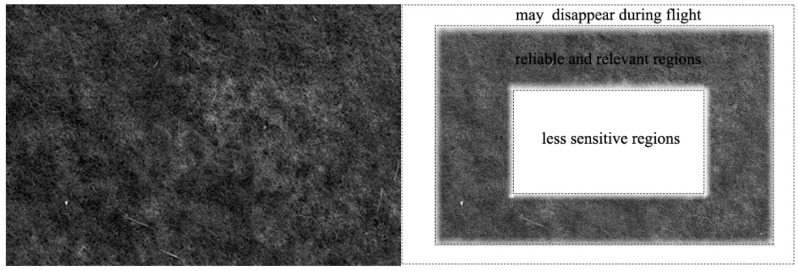
(**Left**) An image captured from a downwards looking camera of the miniature flying ball (MFB); (**Right**) Representation of the regions where measurements are both reliable and relevant.

In order to reduce computing requirements, it is desirable to keep the detection region of images as small as possible. This also indicates that not all of the visual angles in the field of view have the same relationship for flight control. Visual angles pointing at the margin of images correspond to regions that may disappear during flight and, thus, do not require computation. For visual angles close to zero (*i.e*., close to the center of the visual field), the measurable magnitude tends to be zero. These two limits suggest that the region of interest lies around the middle (Figure 3), where measurements are both reliable and relevant for controlling the aircraft.

#### 2.2.1. Feature Extraction

The camera was calibrated to get the intrinsic parameters before use. Then, we can use the parameters to remove the geometrical distortion of the camera and obtain the accurate distances on the image plane.

In the follow-up process, we should find out the corresponding points between subsequence frames and the reference frame. The first step is to extract the features in the frames. There are some common features, such as point, region and contour. Generally speaking, feature point extractions, such as Harris, SUSAN (Smallest Univalue Segment Assimilating Nucleus) and DOG (Difference of Gaussian), are relatively easy in their calculation and are invariable against the rotation, translation and illumination variation.

**Figure 4:**
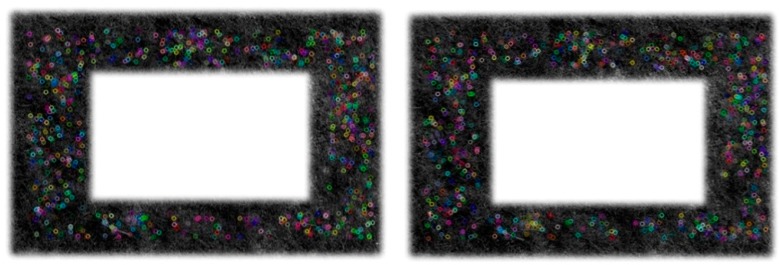
Feature extraction results of the reference frame (**Left**) and one of sequence frames (**Right**).

In the application of our system, the sequence images may change not only in the rotation, translation and illumination variation, but also in the scale and the viewpoint. Therefore, we adopt the SIFT algorithm proposed by Lowe [18], which can deal with these situations. However, the algorithm of SIFT is too time-consuming and is impractical in our system. Therefore, we use the SURF algorithm [19], the improved version of SIFT, to achieve quicker speed and more feature points. The extraction result of SURF is shown in Figure 4.

#### 2.2.2. Feature Matching

**Figure 5:**
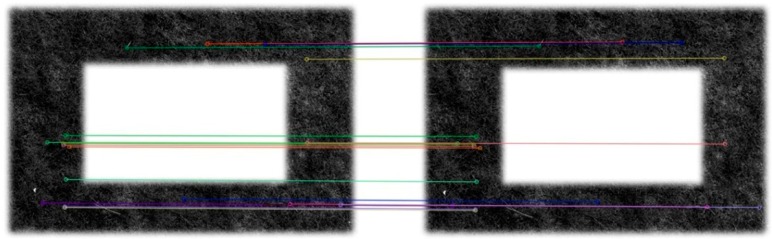
The matching result of two frames. The lines connect the match points between two frames.

After feature extraction, the corresponding points between frames should be matched. Conventional techniques include the correlation coefficient method, the Hausdorff distance method, measurements of similarity, *etc.* We can obtain a multitude of information, such as position, scale, principal orientation and feature vector, about the points when the feature extraction is executed. The feature vectors contain the neighborhood information of the feature points, so we can use the nearest neighbor method to find out the potential matching points to achieve a faster processing speed. The calculation formula can be expressed as follows:

(6) $$D=\left\{\underset{j=1,2,...,N_{2}}{\min}\sqrt{\sum_{i=1}^{k}{\left(f_{ik}-f_{jk}\right)}^{2}}|i=1,2,...,N_{1}\right\}$$

where $N_{1}$ and $N_{2}$ are the number of feature points extracted from two frames, respectively. In order to get a robust matching result, we pick out two minimal candidates denoted as $d_{ij}$ and ${d_{ij}}^{\prime }$. If $d_{ij}\leq \alpha \cdot {d_{ij}}^{\prime }$ (α is set as 0.7 in the experiment), then $d_{ij}$ is considered as the optimal matching point, and the wrong matching can be rejected effectively. The matching results are shown in Figure 5.

#### 2.2.3. Distance of Frames

In order to reduce measurement errors and get accurate data, we use the statistical method to calculate the distance between frames. Assume that we have found N pairs of matching points, and then, we can get the height and x, y variations between two frames from:

(7) $$\{\begin{array}{c} \Delta x=\frac{\sum_{i=1}^{n}x_{i}}{n}-\frac{\sum_{i=1}^{n}x_{i}^{\prime }}{n}\\ \Delta y=\frac{\sum_{i=1}^{n}y_{i}}{n}-\frac{\sum_{i=1}^{n}y_{i}^{\prime }}{n}\\ \frac{h}{h^{\prime }}=\frac{\sum_{i=1}^{n}a^{\prime }b_{i}^{\prime }}{\sum_{i=1}^{n}ab_{i}} \end{array}$$

where $\left(x_{i},y_{i}\right)$ is the coordinate of the feature on the image plane at the target height and $\left(x_{i}^{\prime },y_{i}^{\prime }\right)$ is the coordinate at other moments. $a^{\prime }b_{i}^{\prime }$ and $ab_{i}$ denote the Euclidean distances of each of the two features on the image plane of two moments. Then, we can get the variable (Δx,Δy) in the x and y directions and variable $\frac{h}{h^{\prime }}$ in the z direction.

From Equation (7), we can figure out that the position estimation of the MFB depends on the feature position on the image plane, but is unrelated to the intrinsic parameters of the camera. This is because the position parameters we consider here are relatively variable. The intrinsic parameters of the camera have been eliminated during the calculation. Thus, our method can avoid the measurement error introduced by the calibration error of the camera.

### 2.3. Kalman Filter

A Kalman filter can utilize the basic physics model of the MFB to estimate a dot from its previous position when it is not sensed correctly by the vision system. The Kalman filter will smooth the sensing results and prevent drastic changes in the control commands, which will cause the MFB to become unstable.

In the implementation, when a datum is considered as an outlier, the Kalman filter will respond by modifying the measurement update covariance matrix in order to reduce the confidence in that data. The state vector ($T_{j}$) of the Kalman filter is composed of h and the x and y position variations of the frames, which are being filtered (Equation (8)). The measurement input ($Z_{j}$) of the filter is also h and the x and y position variations of each frame, which make the H matrix of the update equations in the basic Kalman filter equations the identity matrix.

(8) $$T_{j}=\left[\begin{array}{c} h\\ x\\ y \end{array}\right]$$

Three variations are independent of each other, so the equations can be even further transformed, as shown in Equation (10). The equations are then simplified by breaking all matrix operations into single element operations.

(9) $$\begin{array}{l} \hat{u}{\left(k\right)}_{j}=z{\left(k\right)}_{j}-\hat{t}{\left(k\right)}_{j|j+1}\\ \hat{s}{\left(k\right)}_{j}=p{\left(k,k\right)}_{j|j-1}+r{\left(k\right)}_{j}\\ k{\left(k\right)}_{j}=p{\left(k,k\right)}_{j|j-1}s{\left(k\right)}_{j}^{-1}\\ {\hat{t}}_{j|j}=\hat{t}{\left(k\right)}_{j|j-1}+k{\left(k\right)}_{j}\hat{u}{\left(k\right)}_{j}\\ p{\left(k,k\right)}_{j|j}=p{\left(k,k\right)}_{j|j-1}-k{\left(k\right)}_{j}p{\left(k,k\right)}_{j|j-1}\\ k=1,2,3. \end{array}$$

### 2.4. Controller Design

In order to achieve the desired position, three variables h, x and y of the MFB should be controlled with a specific controller. Couplings between the three channels are ignored for simplification. The *h* (height) and the x and y variations are controlled by three off-board PID controllers independently.

For the position x/y of the MFB, the two controllers are similar. P gain is limited to ensure the stability due to the latency of the system. The I term is used to reduce the steady-state errors. The vision data are used to calculate the D terms, since there are no other sensors to measure the speed. Because the field of view (FOV) of the camera is limited, it is necessary to set a threshold value as the output of the PID controller. Otherwise, the corresponding feature points will be out of the FOV, and then, the MFB will be uncontrollable.

For the h-controller, the processing method is basically the same as with x/y. The main difference is that the I term is adjusted to provide enough thrust for the hovering MFB according to different payloads and changing battery status.

## 3. Materials

To validate the proposed hovering control strategy, we ran a series of experiments in a real flying platform developed in our laboratory (Figure 1). In this section, we describe the hardware and software architecture that we used.

### 3.1. Hardware Description

**Figure 6:**
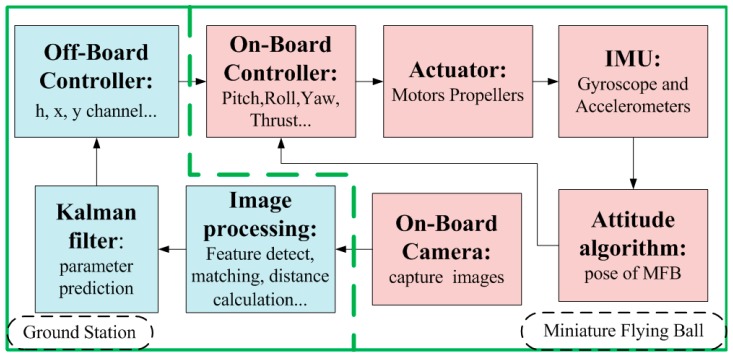
System overview.

The whole system (Figure 6) consists of two parts: the MFB aircraft, consisting of coaxial contra rotating twin rotors, control surfaces, an onboard controller and sensors, such as the camera and IMUs; and the ground station, which receives and processes sensor data, computes the pose of the aircraft, performs the controller strategy and sends the control commands back to the aircraft.

#### 3.1.1. MFB Aircraft

The MFB can perform at 200-Hz control frequency to drive the high-torque DC brushless motors, which enables rapid reaction to changes in the environment. A pair of coaxial rotors spin clockwise and anticlockwise, respectively, which counteracts the rotors’ torque and provide thrust. This is different from similar works, such as the flying robot in [20] and the gimball in [21]. For them, the control surfaces are needed to balance the rotors torque, and this will cause the aircrafts to tilt toward the ground. Our aircraft just needs the thrust of the coaxial contra rotating twin rotors, and it will always keep straight down to the ground during hovering.

The IMU on the MFB aircraft consists of three gyroscopes sensing the angular velocity of each rotation axis and three accelerometers sensing the acceleration of each translation axis. It is used to provide the reference pose of the MFB. Limited by the space and payloads of the MFB, a miniature camera is used in the vision system. The specification of the camera will be discussed in more detail later in the article.

#### 3.1.2. Ground Station

To avoid putting a heavy and powerful computer on the aircraft, images are wirelessly transmitted to a personal computer (PC), through our custom software. An off-board PC (Intel i5, 2 GB RAM) is used for data processing, pose estimation and performing the controller strategy in our system. The aircraft is equipped with the wireless image transmission subsystem and the command transmission subsystem modules, which can transmit image data and control commands at a rate of 20 Hz wirelessly.

### 3.2. Software Architecture

**Figure 7:**
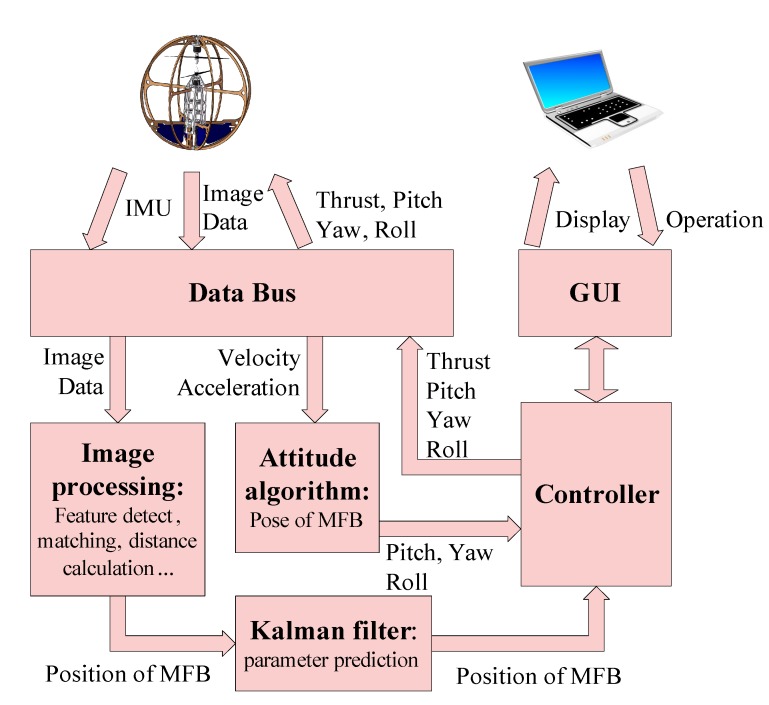
Software architecture.

The main software on the ground station consists of two major parts: vision-based position estimation and a controller. Besides, there are two minor blocks in the entire software structure: the GUI block and the communication block. As shown in Figure 7, the GUI block can provide the interface between the entire system and the user for sensory data displaying, parameter controlling and parameter tuning. The communication block is used to integrate the hardware and software between the aircraft and the ground station.

## 4. Experiments

**Figure 8:**
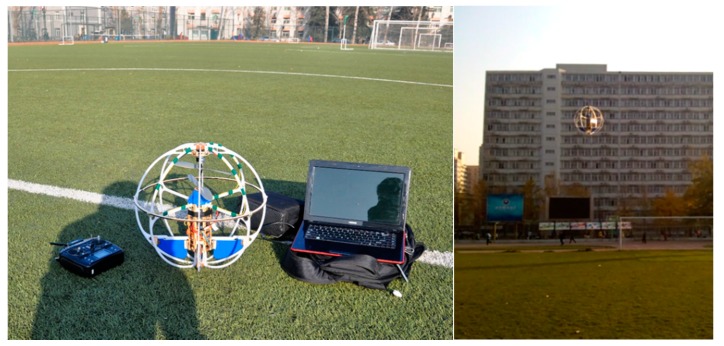
The flying test on the football field.

The performance of the vision-based hovering method is demonstrated using the MFB described in the previous sections. The diameter of the platform is 42 cm, and the total mass is 625 g. The parameters of the selected camera are as follows: the focus length is $8\;\times \;10^{-3}\;m$; the resolution of CCD is 640 × 480; the size of the CCD is ${1/3}^{\prime \prime }$; all algorithm development is in C++, which utilizes the OpenCV library for feature extraction. The experiment environment is a football field shown in Figure 8. The entire system was evaluated in four parts: (1) evaluating the solution of the system; (2) evaluating the static measurement precision of the system; (3) testing the dynamic hovering precision of the MFB; and (4) testing the robustness of the system.

### 4.1. Evaluating the Solution of the System

**Figure 9:**
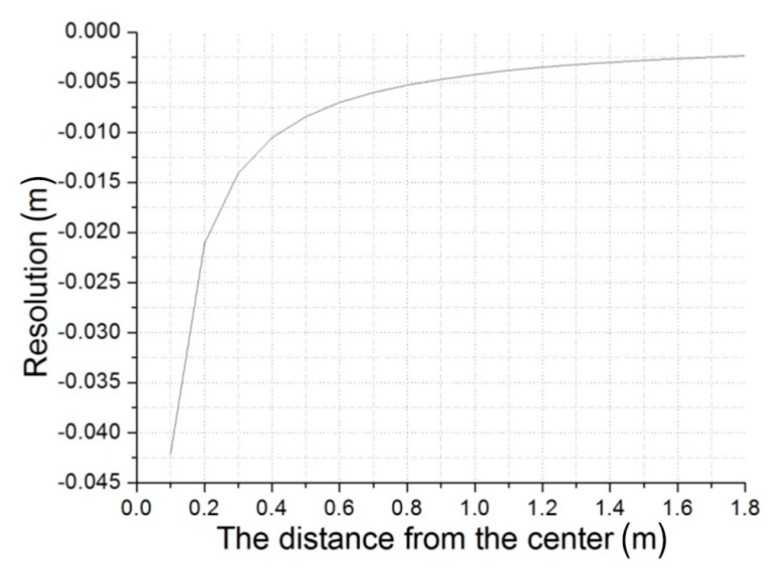
The relation of the resolution with the distance from the center.

Through the derivation of Equation (4) with respect to the length on the image plane, we can get:

(10) $$\Delta h=-\frac{f\cdot l}{x^{2}}\cdot \Delta x=-\frac{h^{2}}{f\cdot l}\cdot \Delta x$$

where Δh represents the height resolution according to the resolution of the CCD Δx. This means that we can sense the minimum change Δh with a specific CCD. Here, assume the height of MFB is set as 3 m. The parameters are substituted into Equation (10), and we can get the relation of the resolution (Δh) with the distance (l) from the center. As can be seen from Figure 9, the resolution near the center (less than 0.8 m) is too coarse, and it will become stable after a certain distance (large than 1.4 m). We can choose the appropriate distance and resolution as we need.

In Equation (10), the $-\frac{h\cdot \Delta x}{f\cdot l}$ is constant for the system. Therefore, the height resolution Δh is linearly related to the height *h*. This means that the higher the aircraft hover, the lower the detection precision will be. Therefore, our method is better applicable within a proper altitude according to the sensitivity of the detector.

### 4.2. Static Measurement Precision of the Vision System of MFB

**Figure 10:**
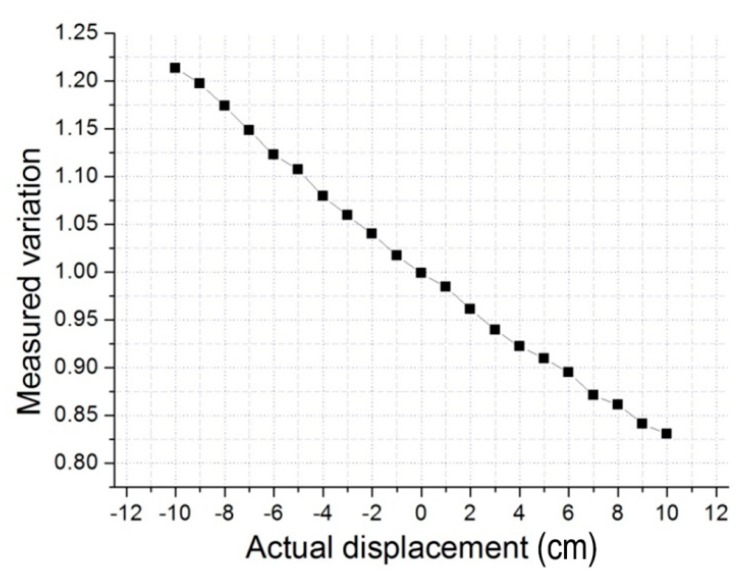
The relation of actual displacement and the measured variation.

In this part, we designed experiments to show the validity of our measurement method. The MFB was hung up by a rope, and its height can be accurately measured by measuring tape. The initial height of the MFB was set at 2 m, and the height could be moved up and down freely. The camera on the MFB looked straight down to the ground, and it took several pictures every time when the height of the MFB changed as planned. According to the schematic diagram presented in Figure 4 and the basic Equation (7), we can get the relationship of $\frac{h}{h^{\prime }}$ and the actual distance variation (Figure 10).

The graph’s horizontal axis shows the actual distance variation from the hovering positon, and the vertical axis shows the $\frac{h}{h^{\prime }}$ calculated by Equation (7). Therefore, in the actual flight, we can figure out the actual distance variation from the hovering positon by $\frac{h}{h^{\prime }}$, which we get from monocular vision. For example, when the $\frac{h}{h^{\prime }}$ equal to 1, this means that the actual positon is at the hovering positon. When the $\frac{h}{h^{\prime }}$ is less than 1, this shows that the actual positon is above the hovering positon. Otherwise, the actual positon is beneath the hovering positon. Therefore, we can maintain the value of $\frac{h}{h^{\prime }}$ equal to 1 by controlling the motion of the MFB to achieve stable hovering.

### 4.3. Dynamic Hovering Precision of MFB

When hovering, the quadrotor is very stable. The 6 DOF of the MFB can be traced by an optical tracking system from NDI Polaris, which uses precise marks to track. The 6 DOF pose of the system obtained by NDI Polaris is used as the standard to inspect the hovering accuracy of the MFB. It can work at a speed of 60 frames per second and recover the position with a precision of around 0.3 mm RMS.

**Figure 11:**
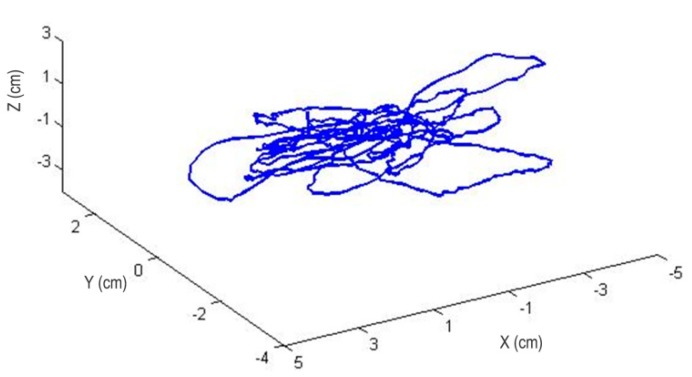
Position error while hovering during 60 s. The RMS value of the position error is 1.54 cm in X, 1.04 cm in Y and 0.92 cm in Z.

**Figure 12:**
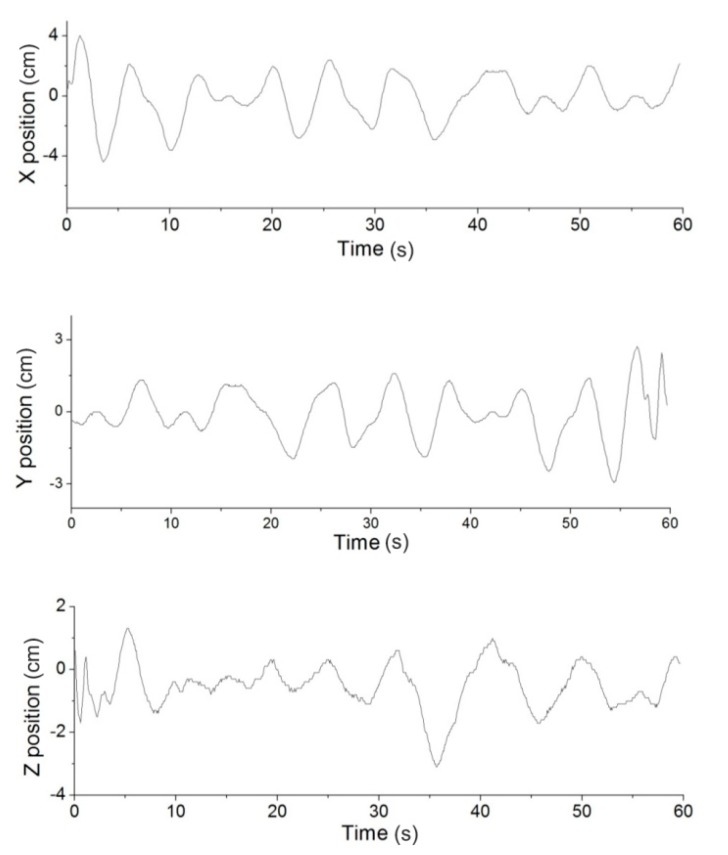
Position error in the X, Y and Z positions. The value remains ±4 cm. The Z position is more accurate than the X and Y positions.

The main experimental processes are as follows: Firstly, the MFB is controlled by the remote control handle to an arbitrary height. Then enable the proposed hovering method is enabled to keep the MFB stable. During the hovering control, NDI Polaris is used to record the trajectory of the MFB. In Figure 11, the height was set at 3 m, and the flight path for a 60-s hover can be recorded and displayed. Overall, the position error during 60 s of hovering has an RMS value of 1.54 cm in the X position, 1.04 cm in the Y position and 0.92 cm in Z position, which yields an absolute RMS error value of 1.36 cm, as shown in Figure 12.

### 4.4. Testing the Robustness of the System

We conduct this experiment to demonstrate the method’s feasibility when the system is affected by a strong disturbance; that is to demonstrate if the MFB can go back to the original hovering altitude when it suffers a sudden change in height, so as to verify the robustness of the system. The experimental setup is the same as that of the previous experiments in Section 4.3. The only difference here is that a sudden height change is employed. To be specific, after the MFB is hovering for a while, we pull it down to certain height (24 cm in this trial) and observe the MFB’s position variation.

**Figure 13:**
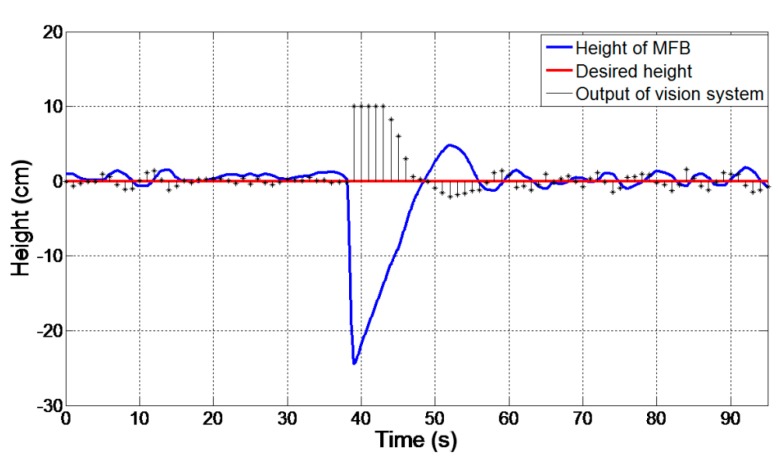
The response to the sudden height change.

The recorded data are shown in Figure 13. The blue line shows the height of the MFB; the red line is the desired height; and the black bars present the corresponding output of the vision system with the proposed method. Note that the outputs are too small to display, so they have been magnified 100-times here. In the experiment, during the first 37 s, the MFB was kept hovering at the expected position with a small deviation. Right at 38 s, it was pulled 24 cm down promptly and released soon. The output of the vision system reached the maximum at this moment. Note that a threshold is set to prevent drastic changes of the output, which will cause the MFB to become unstable. Then, during 38 to 48 s, the MFB returned back to the original height, as expected. In this process, the output decreases with the rising height of the MFB. After 48 s, the ball was hovering around the original height again. The graph above shows that our proposed vision system can always detect the MFB’s orientation and distance and generate negative feedback to control the MFB hovering at the required height.

### 4.5. Discussion

The experiments are based on the assumption that the MFB can always keep straight down to the ground, so that the image planes are completely parallel to each other. Of course, it is impossible to keep the image plane completely parallel without error during the flight. Here, we discuss the error caused by the vibration of the MFB.

**Figure 14:**
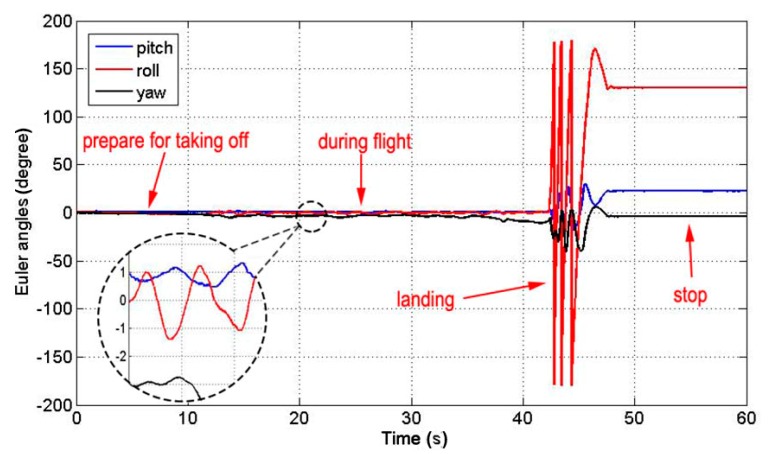
IMU data during one flight.

Figure 14 shows the altitude variation recorded by the IMU of the MFB during one flight with coaxial rotors operating only. At the stage of preparing for taking off, the MFB was kept straight downward, and three Euler angles were initialized to 0. During the flight, the pitch and roll in the horizontal direction varied slightly and kept within a range of less than 1.5°. The yaw in the vertical direction deflected with small angles. When landed, the MFB rolled and bounced to slow down until stopping. This caused the three Euler angles to change drastically. After stopping, the three Euler angles kept steady.

**Figure 15:**
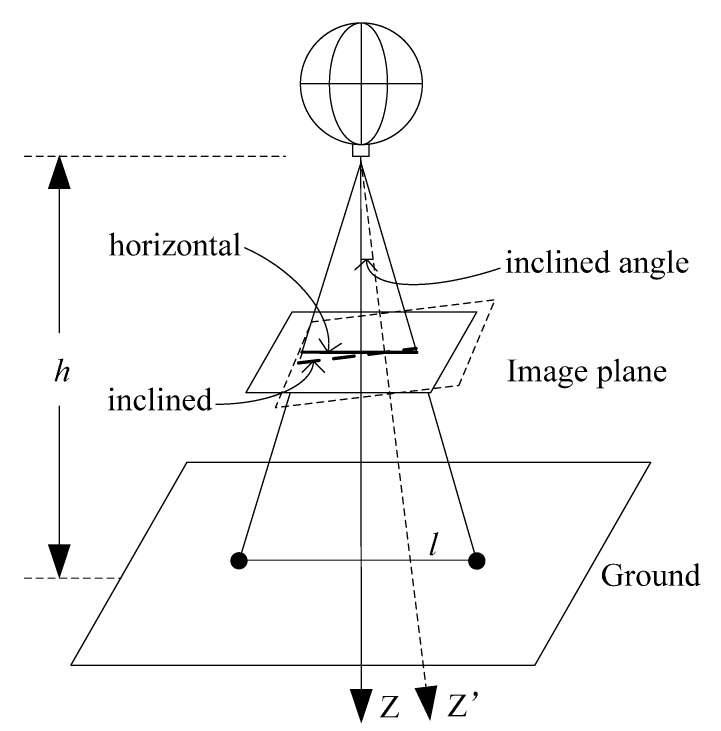
Inclined model of the MFB.

**Figure 16:**
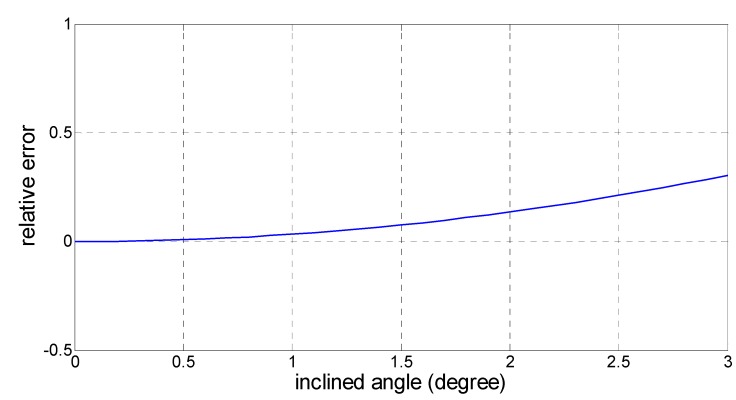
The relationship of the inclined angle with the relative error.

The schematic of the inclined image plane is shown in Figure 15. The relative error caused by the inclined angle to the measurement result can be expressed as:

(11) $$\delta =\frac{\left(\left(\tan\left(\arctan\left(l/h\right)+\Delta \alpha \right)\right)+\left(\tan\left(\arctan\left(l/h\right)-\Delta \alpha \right)\right)\right)-2l/h}{2l/h}\times 100\text{\%}$$

where l is the distance of the feature to the center on the ground, h is the height of the MFB, Δα is the inclined angle and δ is the relative error. Plugging in the conventional value (l=1 m,h=3 m), then we can get the relationship of the relative error δ and the inclined angle Δα, as shown in Figure 16.

In Figure 16, we find that the pitch and roll in the horizontal direction keep within a range of less than 1.5° during normal flight. Then, the relative error caused by the inclined angle will not exceed 0.076%. Therefore, the effect of the inclined angle caused by the tilt of the MFB during flight has little impact on the measurement results. In some other cases, the MFB may be unstable with a great tilt angle caused by the wind or other unpredictable events.

## 5. Conclusions and Future Work

An onboard camera is utilized to measure the position of an MFB to maintain a stable hover. The position variation between the subsequent frames and the reference image can be acquired from analyzing their correspondence points. Then, the measurement is processed by the Kalman filter and sent to the PID controller to control the MFB. The experimental results show that the visual method is able to estimate the aircraft’s positon in unknown environments and to guide the MFB to achieve stable hovering.

Several improvements will be made in the future work. First, we will develop a robust control algorithm, which allows the aircraft to adapt itself to the uncertainty state estimate. Secondly, our work in this paper lacks consideration of bad weather, especially wind, so we plan to expand the functionality of the system by visual techniques in order to adapt to more applications. Thirdly, from Figure 14, we can figure out that the maximum rotation of the MFB is about 10.6° during the 30-s flight. Although this has no effect on our hovering control method, we can derive the rotation of the system from Equation (7) and apply it in the precise control of the MFB in future work.
